# Supplementary material for: Application of Radiomics in Melanoma: A Systematic Review and Meta-Analysis
Source: Cancers (Basel). 2025 Sep 26;17(19):3130. doi: 10.3390/cancers17193130 (PMC12524276; doi:10.3390/cancers17193130)

Figure S1. Forest and Funnel plot of studies in the sub-group analyses.

A) Forest and Funnel plot of studies conducted with CT.

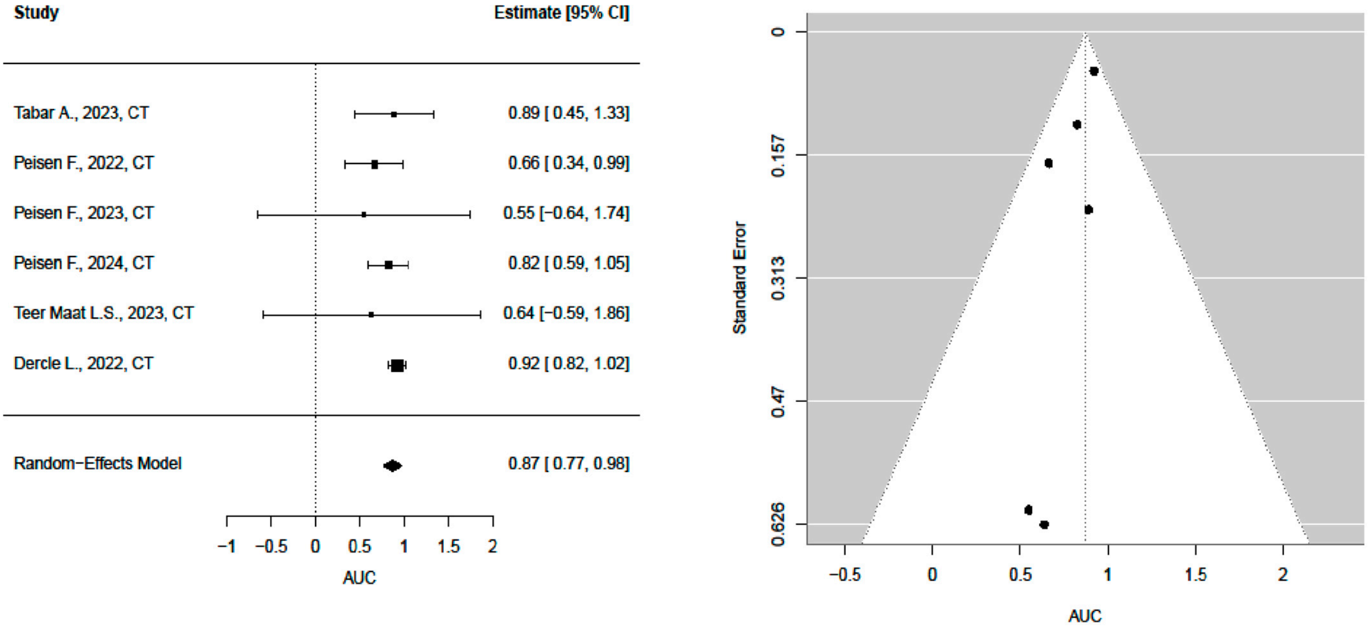

B) Forest and Funnel plot of studies conducted with PET-CT.

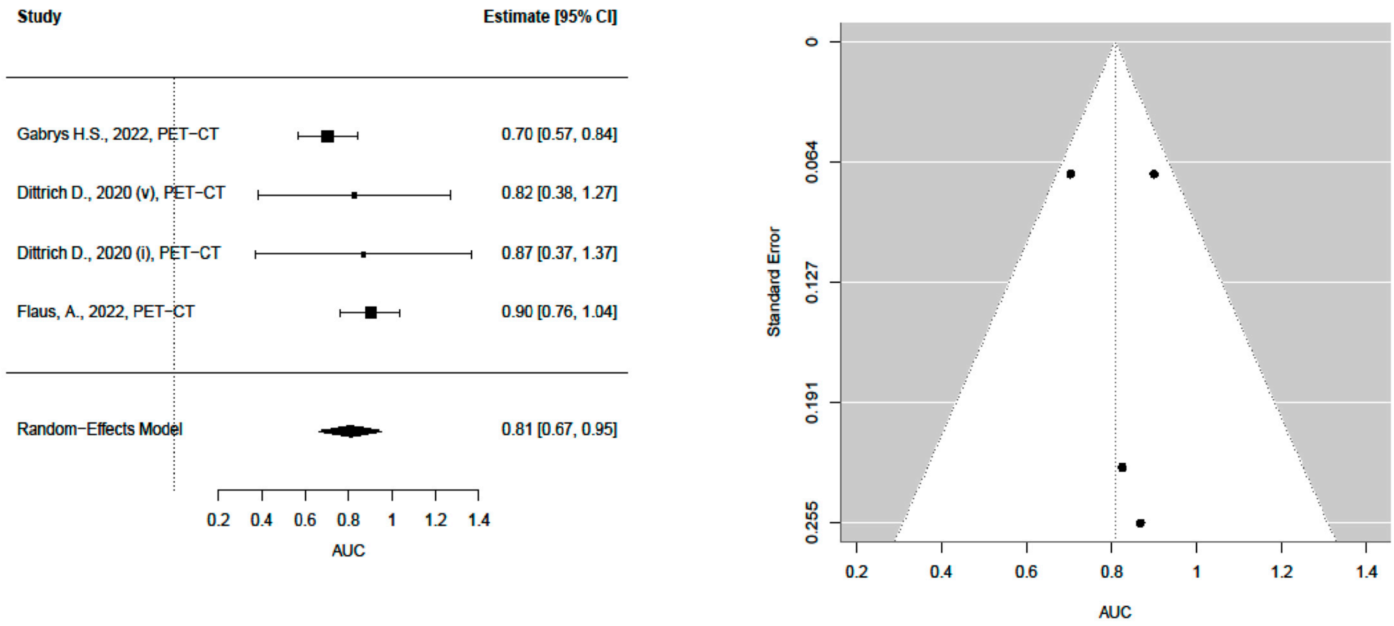

C) Forest and Funnel plot of studies employing cross validation.

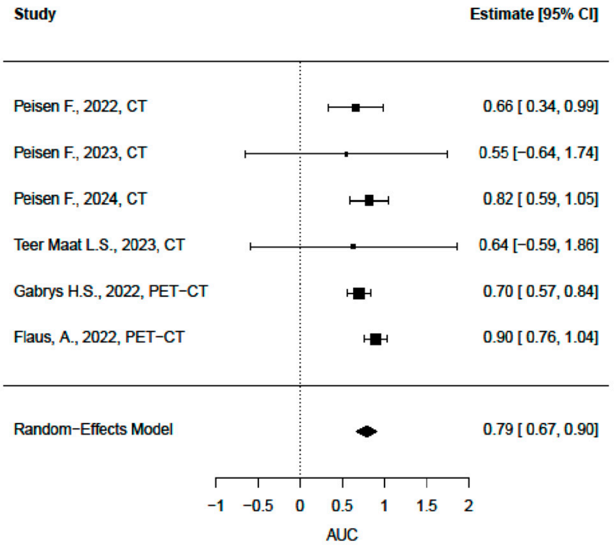

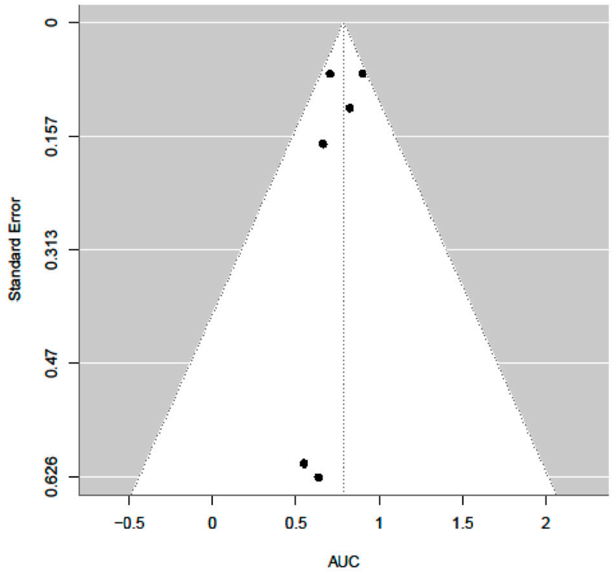

d) Forest and Funnel plot of studies employing internal validation.

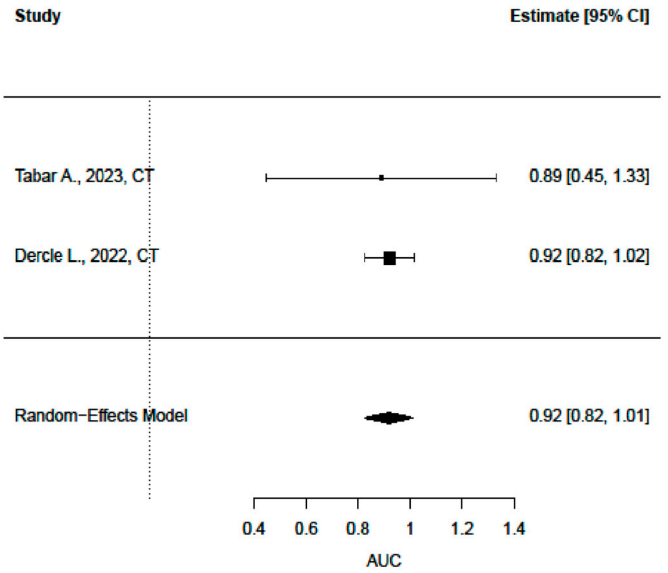

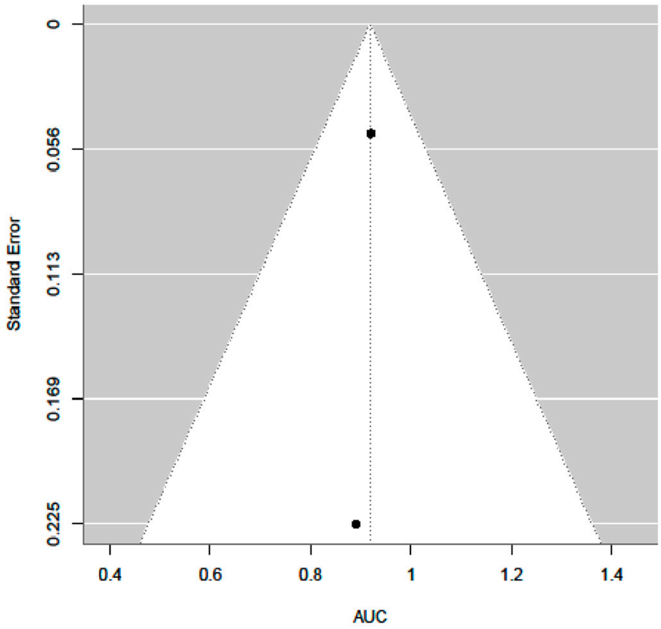

Supplement: Supplementary file 1 [file cancers-17-03130-s001.zip › Figure S1.pdf]
