# Supplementary material for: Application of Radiomics in Melanoma: A Systematic Review and Meta-Analysis
Source: Cancers (Basel). 2025 Sep 26;17(19):3130. doi: 10.3390/cancers17193130 (PMC12524276; doi:10.3390/cancers17193130)
Supplement: Supplementary file 1 [file cancers-17-03130-s001.zip › Table S1.pdf]

Table S1. List of studies included in the systematic review.

Title	Year	DOI
Radiomic analysis of patient and interorgan heterogeneity in response to immunotherapies and BRAF-targeted therapy in metastatic melanoma	2025	10.1136/jitc-2024-009568
Can Delta Radiomics Improve the Prediction of Best Overall Response, Progression-Free Survival, and Overall Survival of Melanoma Patients Treated with Immune Checkpoint Inhibitors?	2024	10.3390/cancers16152669
Correlation Analysis of Apparent Diffusion Coefficient Histogram Parameters and Clinicopathologic Features for Prognosis Prediction in Uveal Melanoma	2024	10.1167/iovs.65.8.3
A CT-Based Radiomics Nomogram Model for Differentiating Primary Malignant Melanoma of the Esophagus from Esophageal Squamous Cell Carcinoma	2023	10.1155/2023/6057196
Machine Learning Improves the Prediction of Responses to Immune Checkpoint Inhibitors in Metastatic Melanoma	2023	10.3390/cancers15102700
Prognostic Value of the Radiomics-Based Model in the Disease-Free Survival of Pretreatment Uveal Melanoma: An Initial Result	2023	10.1097/RCT.0000000000001384
Can Whole-Body Baseline CT Radiomics Add Information to the Prediction of Best Response, Progression-Free Survival, and Overall Survival of Stage IV Melanoma Patients Receiving First-Line Targeted Therapy: A Retrospective Register Study	2023	10.3390/diagnostics13203210
Modeling tumor size dynamics based on real-world electronic health records and image data in advanced melanoma patients receiving immunotherapy	2023	10.1002/psp4.12983
Predicting the BRAF mutation with pretreatment MRI radiomics features for melanoma brain metastases receiving Gamma Knife radiosurgery	2023	10.1016/j.crad.2023.08.012
CT radiomics compared to a clinical model for predicting checkpoint inhibitor treatment outcomes in patients with advanced melanoma	2023	10.1016/j.ejca.2023.02.017
A Decision Support System for the Identification of Metastases of Metastatic Melanoma Using Whole-Body FDG PET/CT Images	2023	10.1109/JBHI.2022.3230060
Metastatic melanoma treated by immunotherapy: discovering prognostic markers from radiomics analysis of pretreatment CT with feature selection and classification	2022	10.1007/s11548-022-02662-8
Radiomics for the noninvasive prediction of the BRAF mutation status in patients with melanoma brain metastases	2022	10.1093/neuonc/noab294
Combination of Whole-Body Baseline CT Radiomics and Clinical Parameters to Predict Response and Survival in a Stage-IV Melanoma Cohort Undergoing Immunotherapy	2022	10.3390/cancers14122992
Principal component analysis of texture features derived from FDG PET images of melanoma lesions	2022	10.1186/s40658-022-00491-x
PET/CT radiomics for prediction of hyperprogression in metastatic melanoma patients treated with immune checkpoint inhibitors	2022	10.3389/fonc.2022.977822

CT texture analysis as a predictor of favorable response to anti-PD1 monoclonal antibodies in metastatic skin melanoma	2022	10.1016/j.diii.2021.09.009
Early Readout on Overall Survival of Patients with Melanoma Treated With Immunotherapy Using a Novel Imaging Analysis	2022	10.1001/jamaoncol.2021.6818
Outcome Prediction at Patient Level Derived from Pre-Treatment 18F-FDG PET Due to Machine Learning in Metastatic Melanoma Treated with Anti-PD1 Treatment	2022	10.3390/diagnostics12020388
Ultra-high-frequency ultrasound and machine learning approaches for the differential diagnosis of melanocytic lesions	2022	10.1111/exd.14330
A Machine learning model trained on dual-energy CT radiomics significantly improves immunotherapy response prediction for patients with stage IV melanoma	2021	10.1136/jitc-2021-003261
The BRAF p.V600E mutation status of melanoma lung metastases cannot be discriminated on computed tomography by LIDC criteria nor radiomics using machine learning	2021	10.3390/jpm11040257
Radiomics biomarkers correlate with CD8 expression and predict immune signatures in melanoma patients	2021	10.1158/1541-7786.MCR-20-1038
Exploring CT Texture Parameters as Predictive and Response Imaging Biomarkers of Survival in Patients with Metastatic Melanoma Treated With PD-1 Inhibitor Nivolumab: A Pilot Study Using a Delta-Radiomics Approach	2021	10.3389/fonc.2021.704607
Textural features in FDG-PET/CT can predict outcome in melanoma patients to treatment with Vemurafenib and Ipilimumab	2020	10.1055/a-1140-5458
Correlating radiomic features of heterogeneity on CT with circulating tumor DNA in metastatic melanoma	2020	10.3390/cancers12123493
Radiomics, Tumor Volume, and Blood Biomarkers for Early Prediction of Pseudoprogression in Patients with Metastatic Melanoma Treated with Immune Checkpoint Inhibition	2020	10.1158/1078-0432.CCR-20-0020
Pilot Study of CT-Based Radiomics Model for Early Evaluation of Response to Immunotherapy in Patients with Metastatic Melanoma	2020	10.3389/fonc.2020.01524
CT texture analysis compared to Positron Emission Tomography (PET) and mutational status in resected melanoma metastases	2020	10.1016/j.ejrad.2020.109242
Value of MR-based radiomics in differentiating uveal melanoma from other intraocular masses in adults	2020	10.1016/j.ejrad.2020.109268
Virtual biopsy using MRI radiomics for prediction of BRAF status in melanoma brain metastasis	2020	10.1038/s41598-020-63821-y
Metabolic biomarker–based BRAFV600 mutation association and prediction in melanoma	2019	10.2967/jnumed.119.228312
Baseline clinical and imaging predictors of treatment response and overall survival of patients with metastatic melanoma undergoing immunotherapy	2019	10.1016/j.ejrad.2019.108688
MRI radiomic features are associated with survival in melanoma brain metastases treated with immune checkpoint inhibitors	2019	10.1093/neuonc/noz141

Metastatic melanoma: pretreatment contrast-enhanced CT texture parameters as predictive biomarkers of survival in patients treated with pembrolizumab	2019	10.1007/s00330-018-5933-x
Predicting response to cancer immunotherapy using noninvasive radiomic biomarkers	2019	10.1093/annonc/mdz108
Correlation between SUV max and CT radiomic analysis using lymph node density in PET/CT-based lymph node staging	2017	10.2967/jnumed.116.179648
Histogram analysis of iodine maps from dual energy computed tomography for monitoring targeted therapy of melanoma patients	2015	10.2217/fon.14.265
Predicting overall survival in patients with metastatic melanoma on antiangiogenic therapy and RECIST stable disease on initial posttherapy images using CT texture analysis	2015	10.2214/AJR.15.14315
Echographic differentiation of intraocular melanomas by computer analysis	1992	10.1111/j.1755-3768.1992.tb04920.x
